# Antibiogram profile of uropathogens isolated at Bahir Dar Regional Health Research Laboratory Centre, Northwest Ethiopia

**DOI:** 10.11604/pamj.2017.26.134.7827

**Published:** 2017-03-10

**Authors:** Awoke Derbie, Derese Hailu, Daniel Mekonnen, Bayeh Abera, Gashaw Yitayew

**Affiliations:** 1 Department of Medical Microbiology, Immunology and Parasitology, College of Medicine and Health Sciences, Bahir Dar University, Bahir Dar, Ethiopia; 2 Bahir Dar Regional Health Research Laboratory Center, Bahir Dar, Ethiopia

**Keywords:** UTI, uropathogens, antimicrobial resistance, Bahir Dar, Ethiopia

## Abstract

**Introduction:**

Antimicrobial resistance among bacteria that cause urinary tract infection (UTI) has been increasing since the introduction of chemotherapy. This study was aimed to assess the types of isolates from patients with UTI and to determine their current antimicrobial susceptibility profile.

**Methods:**

A record based retrospective analysis of bacterial uropathogens processed in the period of January 2012 to December 2014 at Bahir Dar Health Research Laboratory Center (BRHRLC) was determined. According to standard microbiological procedures, midstream urine samples were collected and processed with conventional culture and biochemical tests. Isolates were tested against commonly used antibiotics by Kirby-Bauer disc diffusion methods. Chi-square test was calculated to compare the proportion of bacterial isolates between sex and age and statistical significance was set at p value < 0.05.

**Results:**

Out of 446 patients, female constituted at 299 (67%). Significant bacteriuria was reported on 30.5% (95% CI: 26.4-34.9%) cultures. Of these, 78% (95% CI: 71.0-84.7%) were from females. Age and sex were found associated with significant bacteriuria at p=0.046 and p=0.001 respectively. The most commonly identified isolates were *Escherichia coli*, at 72 (49 %) followed by *Klebshella pneumonia* at 20 (13.6%) and *Pseudomonas aureginosa* at 11 (7.5%). The overall antimicrobial susceptibility profile showed that Trimetoprim-sulphamethoxazole, amoxicillin/clavulanate and ampicillin revealed high level of resistance, at 84(66.7%), 61(79.2%), 106(91.4% respectively. Conversely, 64.2-100% sensitivity rate was documented for ciprofloxacin, gentamycin and pepracillin.

**Conclusion:**

UTI associated with multiple drug resistant bacteria is an important health concern of the study population. Therefore, ongoing surveillance of the types of uropathogens and their up-to-date antimicrobial resistance profile is crucial for better management of patients.

## Introduction

Urinary tract infection (UTI) is an infection occurred anywhere in the urinary system is usually due to bacteria from the digestive tract which ascend the opening of the urethra and begin to multiply to cause infection. In contrast to men, women are more susceptible to UTI, and this is mainly due to their anatomic reason [1–4]. Among the most common infectious diseases, UTIs are commonly encountered by clinicians in developing countries with an estimated annual global incidence of at least 250 million [3] costing in excess of 6 billion dollars [4]. Most UTIs are caused by Gram-negative bacteria like *E. coli*, *Klebsiella spp.*, *P. mirabilis*, *P. aeruginosa*, *Acinetobacter spp.*, and *Serratia spp.* and Gram-positive bacteria such as *Enterococcus spp.* and *Staphylococcus spp* [1, 2]. Drug resistance among bacteria causing UTI has increased since introduction to UTI chemotherapy. In Ethiopia, few studies have been conducted on the prevalence and antimicrobial resistance patterns of UTIs on the general population and specific groups like pregnant women [2, 3, 5, 6]. The etiological agents and their susceptibility patterns of UTI vary in geographical location and through time. Knowledge of the local bacterial etiology and their antimicrobial susceptibility profile is worthy to trace any change that might have occurred in time so that timely updated reference for optimal empirical therapy of UTI can be made [5, 7].

## Methods

**Study area:** The study was conducted at BRHRLC which is one of the new state of the art laboratories in Ethiopia established in 1988. It is the technical arm of Amhara regional health bureau currently providing specialized services (like, MDR-TB culture and molecular laboratory, real time PCR for HIV exposed infants, trachoma elimination research project and quality assurance service). It is giving referral services to Felege Hiwot referral hospital, nearby health centers, private hospitals and clinics.

**Study design and period:** A retrospective review of culture results of patient with presumptive UTI performed in the period of January 2012 to December 2014 at BRHRLC was processed in January, 2015. The age and sex of patients, the kind of bacteria isolated and the antimicrobial susceptibility profile were retrieved from laboratory log book using data extraction sheet.

**Culture and identification:** Patients who did not receive antimicrobials within the previous fifteen days were eligible for the urine culture and this was indicated on request form of the laboratory. Midstream urine samples were collected using sterile plastic containers [8]. Each sample was inoculated on MacConkey agar and blood agar plate (Oxoid Ltd. Bashingstore Hampaire, UK) using 10µl calibrated loops (0.001 ml) and then incubated aerobically at 37oC for 18-24 hours. Each pure colony was counted as colony forming units (cfu). The total cfu were converted in to cfm/ml of urine to check significant bacteriuria. A patient with >10^5^ cfu/ml was considered positive for UTI. For identification of isolates, a panel of biochemical tests had been used based on the gram reaction for gram positives; catalse, cuagulase, bastracin, novobiocin and for gram negative: glucose and lactose fermentation, sulfide-indole-motility, cimon’s citrate, urease lysine iron agar tests] carried out following standard microbiological methods [8–10]. The culture method, identification procedures and reporting system were similar throughout the periods when samples were processed.

**Antimicrobial susceptibility testing (AST):** AST were done on unsupplemented Mueller-Hinton agar (Oxoid Ltd. UK) using Kirby Bauer disk diffusion method (8-9). The antimicrobial agents tested were: Ampicillin (10µg), Amoxicillin/clavulanate (20/10µg), Cefoxitin (30µg) Chloramphenicol (30µg), Ciprofloxacin (5µg), Gentamicin (10µg), Nitrofurantoin (300µg), Norfloxacin (10µg), Pepracillin (100µg) Tetracycline (30µg), and Trimethoprim+Sulphamethazole (25µg) (Oxoid Ltd. Bashingstore Hampaire, UK). Grades of susceptibility were determined after incubation at 370C for 24hs according to Clinical Laboratory Standards Institute (CLSI) [11].

**Statistical analysis:** Statistical analysis was done using Statistical Software Package for Social Sciences (SPSS) version 20 *(IBM Corp. Released 2011. IBM SPSS Statistics for Windows, Version 20.0. Armonk, NY: IBM Corp)*. Chi-square test was calculated to compare the proportion of bacterial isolates with patients’ age and sex and p-value of < 0.05 was considered as significant.

**Ethical considerations:** Ethical clearance was obtained from Amhara Regional Health Bureau Institutional Review Board (IRB) at BRHLC. As the data was collected retrospectively, no patient’s details linked to the patient identity like names were used and confidentiality was maintained.

## Results

**Figure 1:**
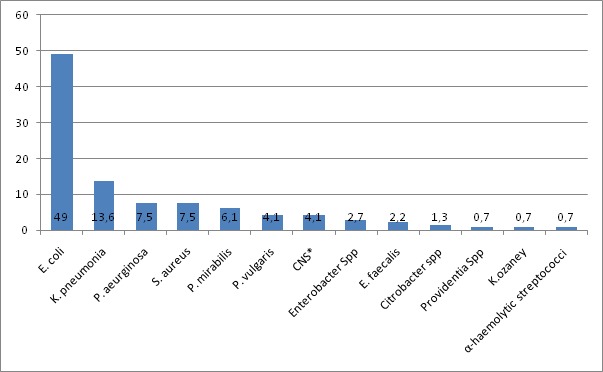
Percentage distribution of isolated bacteria from urine sample in Bahir Dar Regional Research Laboratory Center, December, 2014

During the stated time period, 446 urine cultures were processed. The majority, 299 (67%) urine cultures were from females with female to male ratio of 2.03:1. The median age of the patients was 27 years (range: 47 days to 89 years). Among the total cultures performed, a total of 147 bacterial isolates were identified from 136 (30.5%; 95% CI: 26.4-34.9%) presumptive UTI patients. Among UTI positives, the majority 107 (78.7%; 95% CI: 71.0-84.7%) were females and 76 (55.9%) in the age group of 16-35 years. A urine culture results from females was tend to be more than two times to become positive than males (OR=2.27, 95% CI (1.417-3.628), p=0.001). Similarly, patient’s age was found associated with UTI with (X^2^=9.71, p=0.046). Of the total bacterial isolates, 129 (87.2%) were gram negative rods and the remaining were gram positive cocci (p=0.000) (Table 1 and Table 2). Among bacteriologically confirmed cases, 128 (94.1%) had single isolates and the rest had mixed bacterial infections. The most commonly isolated bacteria were *E. coli*, at 72 (49 %) followed by *K. pneumonia* 20 (13.6%), *P. aureginosa* 11 (7.5%), *S. aureus* 11 (7.5 %) and *P. mirabilis* at 9 (6.1%) (Figure 1). From mixed growth reports *E. coli* was the major partner of the mix (with *P. aureginosa*, *K. pneumonia*, *E. faecalis*, *P.mirabilis*, *P. vulgaris* and *S. aureus*).

**Table 1:** Bacteriological culture results distribution by age and sex in Bahir Dar Regional Research Laboratory Center, December, 2014

	N (%)	N (%)	p-value
**Bacteriological culture results by age**	**Culture negative**	**Culture positive**	
<5	28 (9)	13 (9.6)	**0.046**
5-15	38 (12.3)	7 (5.1)	
16-35	162 (52.3)	76 (55.9)	
36-50	56 (18.1)	20 (14.7)	
>50	26 (8.4)	20 (14.7)	
Total	310 (100)	136 (100)	
**Bacteriological culture results by sex**	**Negative**	**Positive**	**0.001**
Female	192 (61.9)	107 (78.7)	
Male	118 (38.1)	29 (21.3)	
Total	310 (100)	136	

**Table 2:** Antimicrobial resistance pattern of bacterial isolates from urine sample at Bahir Dar Regional Health Research Laboratory Center, December, 2014

Antimicrobial agent	*E. coli*		*K. pneumonia*		*S. aureus*		*P. aeurginosa*		*P. mirabilis*		*P. vulgaris*		CNS		*E. faecalis*		Ent. Spp	
	*#T*	*%R*	*#T*	*%R*	*#T*	*%R*	*#T*	*%R*	*#T*	*%R*	*#T*	*%R*	*#T*	*%R*	*#T*	*%R*	*#T*	*%R*
Ampicillin	64	89.1	17	100	1	100	8	100	7	85.7	3	100	NP	NP	1	0	3	100
Tetracycline	62	66.1	18	27.8	10	60	5	40	7	57.1	3	100	5	40	1	0	3	66.7
Ciprofloxacin	64	64.4	19	21	9	33.3	8	0	6	66.7	3	33.3	5	0	NP	NP	3	66.7
SXT	62	64.5	18	50	8	87.5	7	71.4	7	71.4	3	66.7	3	100	1	0	3	100
Gentamycin	58	27.6	15	40	2	50	7	0	7	57.1	3	100	NP	NP	NP	NP	2	50
Nitrofrantoin	32	25	11	81.8	3	0	NP	NP	5	80	2	50	1	0	1	0	3	33.3
AUG	42	78.6	13	84.6	NP	NP	4	75	5	80	2	50	NP	NP	NP	NP	2	100
Norfloxacin	63	27	18	33.3	NP	NP	7	0	6	50	2	100	NP	NP	NP	NP	2	100
Pepracillin								4	0									
Cefoxitin						7	85.7						6	33.3				

**Key:** #T: number of isolates tested against each antimicrobial agent; %R: percent of isolates resistance to the respective antimicrobial agent; NP: not performed; SXT: Trimetoprim-sulphamethoxazole; AUG: Amoxicillin/clavunate; Ent: Enterobacter. All isolates were not tested for all indicated antimicrobials equally; this is because the agents might stock out at some period. In addition, some antibiotics such as mecillinam and fosfomycin xere not tested due to their regular absence in the laboratory.

With regard to antimicrobial resistance profile of the isolates, the predominant isolate, E. coli, showed significant resistance rates for ampicillin (89.1%), amoxicillin/clavulanate (78.6%) and tetracycline (66.1%). It was found sensitive 72.4-75% to gentamycin, norfloxacin and nitrofrantoin. Similarly, *K. pneumonia* showed resistance rates ranging from 81.8-100% to nitrofrantoin, amoxicillin/clavulanate and ampicillin. However, most of the isolates were sensitive (72.2-79%) to tetracycline and ciprofloxacin (Table 3). The overall antimicrobial susceptibility profile of the isolates showed that Trimetoprim-sulphamethoxazole, amoxicillin/clavulanate and ampicillin revealed high level of resistance, 84(66.7%), 61 (79.2%), 106 (91.4%) respectively. However, 64.2-100% sensitivity rate was documented for ciprofloxacin, gentamycin and pepracillin (Table 3).

**Table 3:** Overall sensitivity and resistance profile of antimicrobial agents tested for bacterial isolates from urine sample, at Bahir Dar Regional Health Research Laboratory Center, December 2014

Antimicrobial agent	Number of antimicrobials tested	Susceptibility pattern		
		Sensitive, N (%)	Intermediate, N (%)	Resistant, (%)
Ampicillin	116	7 (6)	3(2.6)	106 (91.4)
Tetracycline	126	45 (35.7)	7 (5.6)	74 (58.7)
Ciprofloxacin	130	84 (64.6)	4 (3.1)	42 (32.3)
SXT	126	40 (31.7)	2 (1.6)	84 (66.7)
Gentamycin	106	68 (64.2)	2 (1.9)	36 (34)
Nitrofrantoin	64	27(42.2)	9 (14.1)	28 (43.6)
AUG	77	13 (16.9)	3 (3.9)	61 (79.2)
Norfloxacin	110	67 (60.9)	7 (6.4)	36 (32.7)
Pepracillin	4	4 (100)	0	0
Cefoxitin	14	6 (42.5)	0	8 (57.5)

## Discussion

In the present study area, urine culture was one of the frequently requested specimens for culture and AST. This implies that UTI is a common health problem of the society which could partly explained by the result of more than 30% prevalence. Comparable finding was reported by Kibret et al, study [5]. In this study greater than half of the patients were females. In addition among culture positive records, 78.8% were from females. It is also stated in different literatures that females are more prone to UTI than males due to their anatomical nature [1–5, 12]. Peak prevalence of significant bacteriuria/UTI was observed in females with age group of 16-35. This is in agreement with previous studies conducted in Ethiopia, Nigeria, India and Kuwait that reported sexually active females in this age group are at high risk for UTI [3, 5, 13]. Gram-negative bacteria were the dominant isolates (87.8%) compared to gram positives. This is in conformity with different studies elsewhere in the world which indicated that most UTIs are caused by bacteria mainly gram negatives that live in the bowel [1, 2]. Moreover, other studies in Ethiopia, Nigeria and India have demonstrated similar findings [3–5, 14]. This study also showed that *E.coli*, *K. pneumonia*, *P. aureginosa* and *S. aureus* accounted for 77.6% of the isolates. *E.coli* and *K. pneumonia* were the most dominant isolates (49% and 13.6%) respectively. This trend conforms to findings of other studies done in Ethiopia [3, 5] and overseas in India [4]. In contrast, a study in Nigeria [14] has reported that the most common isolated organisms from urine culture were *E. coli* followed by *S. aureus*. Possible explanation for this difference in isolation rate might be related to variation in geographical location and time which is also stated by other studies in the country and abroad [5, 7]. Preceding studies illustrated that UTI is commonly encountered by clinicians’ mainly in developing countries and is one of the major cases for frequent antibiotic use [3, 6].

In the present study, authors have determined current antimicrobial resistance trends of the most predominant bacterial isolates from positive urine culture. *E.coli*, *K. pneumonia* and *P. aureginosas* showed high level of resistant rate (78.6-100%) to amoxicillin/clavulanate and ampicillin. This is slightly in agreement with study by Beyene et al, (3) that demonstrate 100% resistance for ampicillin. Moreover, isolates showed moderate resistance rate to tetracycline, ciprofloxacin and trimetoprim-sulphamethoxazole (64.4- 66.1%). *E. coli* was 72.4-75% sensitive to gentamycin and nitrofrantoin. A study by Asrat et al. also revealed *E. coli* with 89.6 susceptible to nitrofurantoin [15]. In addition (72.2-79%) sensitivity rate to tetracycline and ciprofloxacin was documented for *K. pneumonia*. Furthermore, *P. aureginosas* showed 100% sensitivity to norfloxacin and gentamycin. According to Beyene et al. study [3] all isolates of *E. coli* and *K. pneumonia* were susceptible to ciprofloxacin which is to some extent in variation with the finding of the present study. The possible reason might be related to difference in study population and in additionally literatures also stated that antimicrobial resistance rates among common uropathogens continue to evolve and appear to be increasing to many commonly used agents from time to time [16, 17]. Most of the above mentioned antimicrobials are freely available in local pharmacies and there is an experience that people could purchase and use them without prescription [18]. This might also contribute its share for problem of drug resistance. Generally, this study demonstrated that ciprofloxacin, gentamycin, norfloxacin and pepracillin were the current effective drugs from most uropathogens in the study area.

This study lack extended spectrum betalactamase production status of some the isolates due limited resources in the lab. Moreover, because of the retrospective nature of the study, the authors could not trace patients’ detail clinical and related data, thus the study was exclusively based on bacteriological viewpoint. However, our study is one of the reports that provide current information on the type of bacterial isolates and their antimicrobial resistance profile from urine culture.

## Conclusion

The study showed that UTI is one of the common health problems mainly in females of the reproductive age groups. *E. coli*, *K. pneumonia* and *P. aureginosas* were the predominant bacterial isolates. Most of the isolates showed high levels of antimicrobial resistance to commonly prescribed drugs. Therefore, continued antimicrobial resistance surveillance is indispensable for better management of patients.

### What is known about this topic

- Most cases of UTI are associated with *E. coli*;
- Women are more susceptible to UTI than men due to different factors;
- Bacteria associated with UTI do not have predictable antimicrobial susceptibility profile.

### What this study adds

- Authors depicted the burden of UTI and the associated type of bacterial isolates in the case of Bahir Dar;
- Our study reports current antimicrobial susceptibility profile of the identified bacterial isolates;
- The study finding contributes current figures for large body of literatures on the field of bacterial uropathoges and the increasing trend of antimicrobial resistance.
